# Children’s and Caregivers’ Review of a Guided Imagery Therapy Mobile App Designed to Treat Children With Functional Abdominal Pain Disorders: Leveraging a Mixed Methods Approach With User-Centered Design

**DOI:** 10.2196/41321

**Published:** 2023-04-19

**Authors:** John M Hollier, Tiantá A Strickland, C Michael Fordis, Miranda AL van Tilburg, Robert J Shulman, Debbe Thompson

**Affiliations:** 1 Texas Children's Hospital Houston, TX United States; 2 Department of Pediatrics Baylor College of Medicine Houston, TX United States; 3 The Center for Collaborative & Interactive Technologies Baylor College of Medicine Houston, TX United States; 4 Department of Internal Medicine Marshall University Huntington, WV United States; 5 School of Social Work University of Washington Seattle, WA United States; 6 Department of Gastroenterology and Hepatology University of North Carolina Chapel Hill, NC United States; 7 Graduate Medical Education Cape Fear Valley Hospital Fayetteville, NC United States; 8 Children's Nutrition Research Center Agricultural Research Service United States Department of Agriculture Houston, TX United States

**Keywords:** guided imagery therapy, guided imagery, psychotherapy, disorders of gut-brain interaction, functional abdominal pain disorders, pediatric, pain, mobile app, mobile health, mHealth, mixed methods research, usability, gamification, user-centered design, guided image therapy, app prototype, prototype, feedback, children, child, youths, caregiver, mobile phone

## Abstract

**Background:**

Functional abdominal pain disorders (FAPDs) are highly prevalent and associated with substantial morbidity. Guided imagery therapy (GIT) is efficacious; however, barriers often impede patient access. Therefore, we developed a GIT mobile app as a novel delivery platform.

**Objective:**

Guided by user-centered design, this study captured the critiques of our GIT app from children with FAPDs and their caregivers.

**Methods:**

Children aged 7 to 12 years with Rome IV–defined FAPDs and their caregivers were enrolled. The participants completed a software evaluation, which assessed how well they executed specific app tasks: opening the app, logging in, initiating a session, setting the reminder notification time, and exiting the app. Difficulties in completing these tasks were tallied. After this evaluation, the participants independently completed a System Usability Scale survey. Finally, the children and caregivers were separately interviewed to capture their thoughts about the app. Using a hybrid thematic analysis approach, 2 independent coders coded the interview transcripts using a shared codebook. Data integration occurred after the qualitative and quantitative data were analyzed, and the collective results were summarized.

**Results:**

We enrolled 16 child-caregiver dyads. The average age of the children was 9.0 (SD 1.6) years, and 69% (11/16) were female. The System Usability Scale average scores were above average at 78.2 (SD 12.6) and 78.0 (SD 13.5) for the children and caregivers, respectively. The software evaluation revealed favorable usability for most tasks, but 75% (12/16) of children and 69% (11/16) of caregivers had difficulty setting the reminder notification. The children’s interviews confirmed the app’s usability as favorable but noted difficulty in locating the reminder notification. The children recommended adding exciting scenery and animations to the session screen. Their preferred topics were animals, beaches, swimming, and forests. They also recommended adding soft sounds related to the session topic. Finally, they suggested that adding app gamification enhancements using tangible and intangible rewards for listening to the sessions would promote regular use. The caregivers also assessed the app’s usability as favorable but verified the difficulty in locating the reminder notification. They preferred a beach setting, and theme-related music and nature sounds were recommended to augment the session narration. App interface suggestions included increasing the font and image sizes. They also thought that the app’s ability to relieve gastrointestinal symptoms and gamification enhancements using tangible and intangible incentives would positively influence the children’s motivation to use the app regularly. Data integration revealed that the GIT app had above-average usability. Usability challenges included locating the reminder notification feature and esthetics affecting navigation.

**Conclusions:**

Children and caregivers rated our GIT app’s usability favorably, offered suggestions to improve its appearance and session content, and recommended rewards to promote its regular use. Their feedback will inform future app refinements.

## Introduction

### Background

Functional abdominal pain disorders (FAPDs) are a group of chronic abdominal pain conditions (eg, irritable bowel syndrome) that cannot be clearly attributed to an organic etiology [1]. These disorders, now often referred to as disorders of gut-brain interaction, affect approximately 15% of school-age children, adolescents, and adults worldwide [1-3]. Children with FAPDs have a lower health-related quality of life than their healthy peers and those with other organic gastrointestinal diseases such as inflammatory bowel disease [4]. The pathophysiology of FAPDs is often characterized as perturbations of the physiological, genetic, environmental, and psychosocial factors that affect the bidirectional communication between the gut and brain [5].

Treatment for children with FAPDs is available through pharmacological and nonpharmacological means, but of these options, psychological therapies such as cognitive behavioral therapy and hypnosis have greater clinical evidence supporting their efficacy [5-7]. Unfortunately, the logistics for obtaining psychological interventions for FAPDs are often marred by barriers. There is an insufficient labor pool of trained mental health providers to treat the many children affected by FAPDs [8]. Furthermore, the need for repeated therapy sessions and inadequate mental health insurance coverage often impede optimal care [9,10]. As an alternative delivery modality, one research group reported that prerecorded guided imagery treatment—a form of self-hypnosis—delivered remotely through CDs was efficacious for children with FAPDs [11]. In another study, audio-delivered hypnosis was not inferior to therapist-delivered hypnosis [12,13], indicating that this type of treatment is likely effective.

This literature on remotely delivered therapy motivated us to develop a guided imagery therapy (GIT) mobile app prototype as a readily accessible means to treat children with FAPDs. Our previous predevelopment formative research with children with FAPDs and their mothers revealed a strong interest in this novel delivery approach, as both groups were intrigued by a nonpharmacological, efficacious treatment for abdominal pain symptoms [14].

### User-Centered Design

One of the approaches to app development is user-centered design, which focuses on creating a product that meets the needs of the targeted users by capturing and communicating their needs to researchers and developers [15]. User-centered design can be costlier to develop but will likely produce a product that meets users’ needs and requires less technical assistance on the part of the user [16,17]. The user-centered design approach incorporates end users’ input throughout development and typically addresses the essential qualities of the app, such as usability, which is a measure of the ease by which an app can be learned and used [18].

Guided by user-centered design, this study’s objective was to obtain feedback from children with FAPDs and their caregivers about our GIT app by using multiple research methods. These stakeholders’ critiques will inform the refinement of the GIT app before future efficacy testing.

## Methods

### Ethics Approval

The institutional review board at Baylor College of Medicine approved this study protocol (H-45050). After fully explaining our study protocol, the caregivers and their children documented on paper their informed consent and assent for participation in the study, respectively. Informed consent was obtained before any study data were collected. We explained that study participation was completely voluntary and that they could withdraw from the study at any time. The study involved listening to a guided imagery session, and there was a risk of transient headache and anxiety. The possibility of a breach of confidentiality was mentioned in the consent form. This risk was minimized by deidentifying study participant data, storing data on secure institutional servers, and allowing data access to research staff only. The children and caregivers were compensated a total of US $40 for their participation.

### Participants

Patients aged 7 to 12 years meeting the criteria for an FAPD [1] and their caregivers were recruited from a single ambulatory health care system with 52 ambulatory care clinics in metropolitan Houston, Texas. We posted advertisements in the ambulatory care clinics, and interested patients contacted our research team for screening. We also identified prospective participants through our institution’s electronic medical record by querying ambulatory office visits for FAPD. We then sent recruitment letters to the prospective participants’ homes after receiving permission from their treating provider. All potential participants completed a prescreening questionnaire and agreed to have their child’s medical record screened for inclusion and exclusion criteria.

We excluded participants with prior abdominal surgeries, comorbid conditions associated with abdominal pain (eg, cystic fibrosis), autism, significant developmental delay, psychosis, prior experience with psychological therapies to treat abdominal pain, any alarming symptoms that warranted further medical evaluation (eg, weight loss), or not fluent in English.

The children and their caregivers completed the pediatric Rome IV questionnaire to confirm the presence of an FAPD and determine the subtype (eg, functional dyspepsia) if present [19]. The children whose ages were ≥10 years completed the Rome IV questionnaire independently. The children whose ages were <10 years were not administered the self-report Rome IV questionnaire. All the caregivers completed the parent-report version of the Rome IV questionnaire. The participants were enrolled in the study if the self-report or parent-report Rome IV questionnaire confirmed the presence of at least 1 FAPD. To complete the remaining aspects of the study, the children and their caregivers were taken to separate rooms without communication.

### Mixed Methods Study Design

Our mixed methods approach had a convergent parallel design, which gathered quantitative data (a validated usability questionnaire and software evaluation summaries) and qualitative data (child and caregiver interviews) to investigate the GIT app’s usability [20]. The results from each method were compared and integrated to assess the overall usability of the app from the children’s and caregivers’ perspectives.

### Data Collection

#### Demographic Questionnaires

The caregivers completed a demographic questionnaire that captured the children’s age, sex, and self-reported race and ethnicity. The caregivers also provided their sex, self-reported race and ethnicity, marital status, educational level, and household income.

#### GIT Mobile App Prototype Recorded Software Evaluation

**Figure 1:**
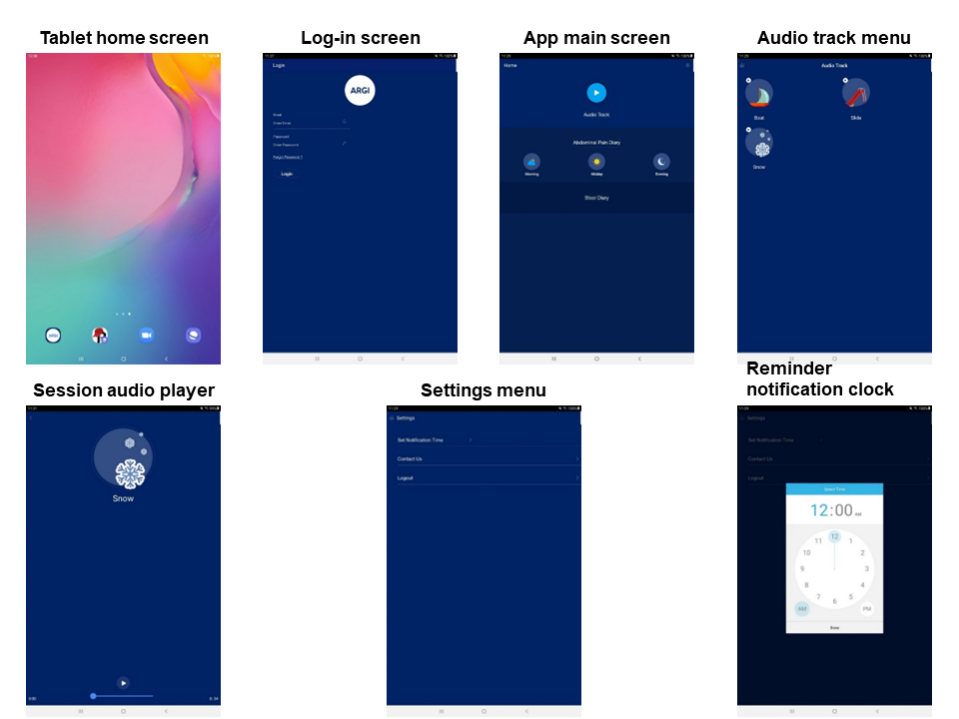
Screenshots of the guided imagery treatment mobile app.

Before the testing session, the prototype app was installed on 2019 Samsung Galaxy Tab A 10.1 tablets (Samsung Group) running on Android 11. The dyads presented in person for a single visit that lasted approximately 3 hours. Each participant (child and caregiver) went to a separate room with a research team member, where they participated in the software evaluation. At the initiation of the testing session, the study participants were given a task list by a research team member. In the task list, each participant was asked to complete the following actions on the app: open the app, log-in with a preassigned email and password, open the submenu containing the GIT sessions, select a sample session, play a sample session, set a reminder notification for 7:30 PM, and exit the app (Multimedia Appendix 1). Screenshots of the GIT app named *ARGI* (Audio Recorded Guided Imagery) are presented in Figure 1. A research team member observed the caregiver or child completing the task list while sitting next to them and was available to answer questions and provide assistance. After opening the sample therapy session, the caregiver and child listened to a sample 8-minute-and-30-second session that previously demonstrated efficacy in a pediatric clinical trial (provided by the author MALvT) [11]. The sample included an induction phase, and all therapeutic suggestions for improvement were removed. The software evaluation session was digitally recorded and later viewed by the staff.

#### System Usability Scale Usability Measure

After completing the software evaluation, we used a validated survey to capture the GIT app’s usability. Both children and their caregivers individually completed the System Usability Scale (SUS) questionnaires (Multimedia Appendix 2 and Multimedia Appendix 3, respectively) [21,22]. This 10-item validated survey has been used to assess the overall usability of various technologies, including hardware, software, mobile devices, websites, and apps [23]. The survey was modified to enhance comprehension in children who tested our GIT app. For example, we changed the wording “complex” to “hard to use” (item 2) and “cumbersome” to “very hard” (item 8; refer to Multimedia Appendices 2 and 3). The SUS uses a 5-point Likert scale to assess items: 1=strongly disagree, 2=disagree, 3=neutral, 4=agree, and 5=strongly agree. Using the SUS scoring instructions, we transformed the response item scores and subsequently calculated the overall SUS score. The score contribution of odd-numbered items was transformed by subtracting 1 from the scale scores. The score contribution of even-numbered items was transformed by subtracting the item response from 5. These 10 transformed item scores were then summed and multiplied by 2.5 to obtain the overall SUS score. The overall SUS scores can range from 0 to 100, with higher scores indicating higher usability [21]. A usability score of 68 equates to the 50th percentile [24].

#### Interviews

After completing the task list and listening to the sample guided imagery session, the children and their caregivers participated in separate interviews. Trained interviewers conducted the interviews using a semistructured script (Multimedia Appendix 4 for the children and Multimedia Appendix 5 for the caregivers). The interviews were designed to last ≤1 hour each. Probes and prompts were used to expand and clarify the responses. The interviews aimed to explore perceptions regarding usability, elicit critiques and preferences for the sample session, and identify factors that may affect the use of the GIT app by children with FAPDs. All interviews were audio recorded, transcribed verbatim, and reviewed for accuracy before analysis. The corrected transcripts served as the unit of analysis.

### Data Analysis

#### SUS Analysis

The overall SUS scores were calculated separately for each child and caregiver, and the average score was computed for each group. Sample variability and reliability were assessed by calculating each group’s SD and Cronbach α, respectively. Calculations were conducted using SPSS Statistics (version 27; IBM Corp) for Windows (Microsoft Corp). Results were provided as mean (SD) and Cronbach α. SUS scores were assigned qualifying adjectives based on their mean value per Bangor et al [23].

#### GIT App Software Evaluation Analysis

The research team members reviewed the video recordings of the software evaluation sessions to assess whether the participants could complete the 5 assigned tasks. Difficulty with a task was defined as a participant asking the research team member for assistance with a particular task or failing to complete a specific task. Using a checklist, the research team reviewed all videos and tallied each participant’s task difficulties (yes or no). The frequency of specific task difficulties was recorded separately for the children and caregiver groups.

#### Interview Analysis

The reviewed and corrected transcripts were analyzed by trained coders. Before the analysis, a codebook containing a priori codes and corresponding definitions was created [25]; the a priori codes were based on the questions in the interview script. During the analyses, additional codes and corresponding definitions were identified by the coders based on the participant responses. Separate codebooks were maintained for the caregiver and child interviews.

Before full-scale coding, the coders initially coded 2 transcripts to ensure consistency in the application of the codebook. Then, they met to discuss the application of the codes and resolve coding differences (JMH and TAS). Once the coders were confident that the codebook was being applied consistently, the remaining transcripts were independently coded using a hybrid thematic analytic approach [25]. Throughout the coding process, routine meetings were held to discuss coding and resolve discrepancies. The child and caregiver transcripts were coded and analyzed separately. Coding was performed using NVivo (version 10, QSR International) for Windows.

### Mixed Methods Integration

After the analysis, the findings derived using each method were compared using methodological triangulation [20,26] to determine whether the study findings converged, complemented, or contradicted one another [26,27]. The data were then integrated and summarized to assess usability [28].

## Results

### Demographics

In total, 16 patient-caregiver dyads were enrolled in the study and completed all study phases. The demographics of the children and their caregivers are summarized in Table 1. The average age of the children was 9.0 (SD 1.6) years, and most children (11/16, 69%) were female (Table 1). Our sample primarily comprised non-Hispanic White participants, but the overall sample was racially and ethnically inclusive (Table 1). Of the 16 child participants, 1 (6%) child’s responses did not meet the Rome IV criteria for an FAPD diagnosis, but their caregiver’s responses to the parent-report Rome IV questionnaire did reveal that they met the criteria (Table 1). Similarly, 13% (2/16) of caregivers’ responses did not meet the criteria for an FAPD diagnosis for their children, but their children’s responses met the criteria (Table 1).

**Table 1:** Descriptive statistics of children with functional abdominal pain disorders (FAPDs) and their caregivers (n=16 dyads).

Characteristics				Sample proportion, n (%)
**Child participants**				
	**Age range (years)**			
		7-8	7 (44)	
		9-10	6 (38)	
		11-12	3 (19)	
	**Sex**			
		Female	11 (69)	
		Male	5 (31)	
	**Self-reported race and ethnicity**			
		Non-Hispanic White	9 (56)	
		Hispanic White	3 (18)	
		Non-Hispanic Black	2 (13)	
		Non-Hispanic Asian	2 (13)	
	**FAPD types^a^**			
		Functional dyspepsia	5 (83)	
		Irritable bowel syndrome	1 (17)	
		Functional abdominal pain—not otherwise specified	2 (33)	
		Multiple disorders identified	2 (33)	
		No disorders identified	1 (17)	
**Caregiver participants**				
	**Sex**			
		Female	14 (87)	
		Male	2 (13)	
	**Self-reported race and ethnicity**			
		Non-Hispanic Asian	1 (6)	
		Non-Hispanic Black	2 (13)	
		Non-Hispanic White	11 (69)	
		Hispanic White	2 (13)	
	**FAPD types**			
		Functional dyspepsia	14 (88)	
		Abdominal migraine	2 (13)	
		Multiple disorders identified	2 (13)	
		No disorders identified	2 (13)	
	**Median household income (US $)**			
		15,000-29,000	1 (6)	
		30,000-49,999	2 (13)	
		≥50,000	12 (75)	
		Prefer not to say	1 (6)	

^a^6 children were eligible to complete the Rome IV questionnaire.

### Mobile App Software Evaluation

All 16 dyads completed the software evaluation. The evaluation’s average duration (minutes) for the children and their caregivers was 3.6 (SD 1.2) and 2.5 (SD 1.1), respectively, excluding the sample guided imagery session playback. Staff review of the video recordings of the evaluation revealed that all the children (16/16, 100%) could open the app, open the audio track submenu, and start a sample GIT session. However, 6% (1/16) of children had difficulty logging into the app using an email address and password, and 75% (12/16) of children had difficulty setting the reminder notification time feature, which alert users to complete a guided imagery session by programming an audio alarm.

All the caregivers (16/16, 100%) could open the app, log into the app using an email address and password, open the audio track submenu, and start a sample session. However, 69% (11/16) of caregivers had difficulty setting the reminder notification time. Both children and caregivers had difficulty locating this feature’s settings within the app; it was located on the App Main Screen denoted by a gray gear icon (Figure 1, in the top-right corner).

### SUS Results

Because we made a few word choice–related changes to adjust the SUS for children, we calculated the internal consistency of the new scale. The Cronbach α for the children and caregiver groups were .77 and .88, respectively. The children’s average overall SUS score was 78.2 (SD 12.6), and the caregivers’ average score was 78.0 (SD 13.5) out of 100 points. Both mean SUS scores range between “good” and “excellent” [23].

### Participant Interview Results

#### Overview

**Figure 2:**
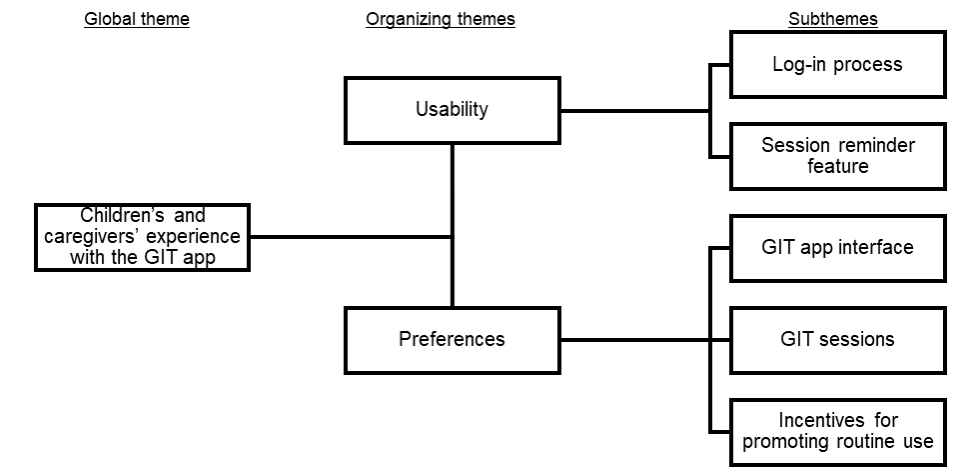
Thematic network of the experiences of the children with functional abdominal pain disorders and their caregivers with the guided imagery therapy (GIT) app for treating pediatric functional abdominal pain disorders.

The qualitative interviews focused on the participants’ overall experience with our GIT app by exploring their perceptions of app usability and their preferences. This global theme and these organizing themes formed the core of our thematic network for both children and caregivers (Figure 2) based on similar study goals and structure of the interview guides for both groups. The following qualitative analyses elaborated on these organizing themes and their subthemes (Figure 2).

#### Children’s Experience Using the GIT App

The children’s experiences with the GIT app were organized by usability and preferences, as shown in Figure 2. Representative quotes, identified by the child’s age, race and ethnicity, and birth sex, are provided to support the subthemes.

##### Usability

This organizing theme summarized the feedback of the children with FAPDs regarding the ability to navigate and perform specific tasks within the GIT app. They found the GIT app’s usability favorable. Many children felt that the GIT app was easy to use and responded as expected to commands. The children’s specific comments primarily focused on the app’s log-in process and setting the session reminder feature.

###### Log-in Process

This subtheme summarized the comments regarding the functionality of the GIT app’s log-in screen. The participants generally did not have an issue logging into the app. The children thought that the log-in process using a username and password was easy:

Like it was just like, like getting onto it. So it was pretty easy like logging in, and like to getting like to where I need to go.

Non-Hispanic White male child aged 11 years

###### Session Reminder Feature

The children revealed that they would like to be prompted to listen to a session through their electronic devices. They preferred a pop-up notification on their devices and SMS text messages as alerts for session reminders. However, the current location for setting the session reminder feature within the GIT app was difficult to locate. This was noted by a child as follows:

The settings was, the only thing I never...notifications, usually aren’t in setting boxes, they’re usually a little box for notifications, so that was a hard part about it, finding stuff [the reminder notification setting screen].

Hispanic White female child aged 9 years

##### Preferences

The GIT app preference organizing theme summarized critiques related to the app’s components, including its interface esthetics and session content. This theme also summarized the preferred incentives for promoting routine engagement with the app.

###### GIT App Interface

This subtheme summarized the children’s thoughts about the GIT app’s visual appearance. The children agreed that changes should be made to the appearance of the GIT app, but there was a lack of consensus on what should be changed. They liked the current pictures and colors, including the blue tones currently used in the app. Children also liked that the app was “plain” and “simple.” They suggested modifying various images, including a baby blue background, and adding a grassy meadow with trees and water, fish, islands, smiling real-life kids with their thumbs up, butterflies in the woods, birds, and a monkey. A couple of children thought that adding animation to the image on the guided imagery session player screen would be desirable. A few children thought that the app’s appearance looked “good” or “nice” and recommended adding colors for a more “exciting” and “surprising” background:

If the app [background] did have colors, it would be light colors, calm colors. And not too bright, hot and neon. It’d probably be like this greyish color, maybe like a light blue, pink, green, yellow, calm colors that just make you relax and don’t make you stressed.

Non-Hispanic White female child aged 10 years

Alternatively, the children identified several undesirable characteristics regarding the app’s appearance. They thought the app icons, such as the gear image used for the settings option, were too small. Finally, they thought that the font style was satisfactory but that the font size was too small on their tablet screens.

###### Content of the GIT Sessions

This subtheme captured the children’s thoughts about the ideal sessions that should be incorporated into the app. The children critiqued the sample session and provided further information about what they would generally desire in sessions.

They thought that the guided imagery sample session was relaxing and liked the snow sledding narrative. They also thought that the female narrator’s voice was calming:

Yes, because it [guided imagery session] relaxes you a lot, and it—relaxing is very good for your stomach pains. Just something simple like going outside and look at the stars, that’s actually very relaxing for your stomach, but if you don’t have that kind of time, just listen to the audio track.

Non-Hispanic Asian female child aged 10 years

The children generally recommended session settings that include animals, such as dogs, cats, elephants, and horses. Outdoor adventures at beaches, jungles, playgrounds, rainforests, and forests were ideal. They also liked the idea of swimming and playing in the sun during a session. Moreover, they suggested adding sounds to the guided imagery sessions. They specifically mentioned nature sounds such as those of ocean waves, rain, and birds chirping.

The children stated that they would not recommend topics related to specific types of animals, violence, or scary scenarios. Particular animals such as spiders, snakes, tigers, sharks, or lions were not recommended for sessions because they were characterized as “scary.” The children did not recommend sessions with scary or loud noises or crimes such as stealing, burglary, and kidnapping. Finally, they did not want sessions located in dark settings.

No clear consensus emerged regarding the ideal session length. Most children preferred a length of 5 to 10 minutes for sessions. The responses ranged from 54 seconds to 30 minutes.

###### Incentives for Promoting Routine Use

This subtheme captured the children’s preferred incentives for promoting the use of the GIT app. The children agreed that rewards would increase their motivation to regularly listen to the sessions within the app. They liked the concept of receiving tangible rewards such as money, food, and toys:

Yeah, coupons to get food, free coupons sent to my phone to get food or points on some food apps, like the Chick-Fil-A app or the Wingstop app.

Non-Hispanic Black male child aged 12 years

The children also mentioned that encouraging messages provided through the app and those provided externally (eg, mother’s encouragement) would motivate their use. They endorsed that receiving intangible points or coins for completing sessions would be desirable. They also suggested that unlocking new sessions could be another incentive for participation.

#### Caregivers’ Experience With the GIT App

The caregivers’ experiences with the GIT app were classified into 2 themes: usability and preferences. Representative quotes from the caregivers are presented in the subsequent sections along with the subtheme descriptions (Figure 2).

##### Usability

This organizing theme captured comments regarding the caregivers’ ability to navigate and perform specific tasks within the GIT app. The caregivers found the GIT app to have acceptable usability, as they could perform most app-specific tasks without difficulty. However, they endorsed difficulty setting the session reminder feature and provided additional comments about logging into the app.

###### Log-in Process

Overall, the caregivers did not have difficulty logging into the app. They thought that logging in using a username and password was easy:

The log-in screen is clear, you know they’re not going to, I mean either my daughter...they’re already logging in at school on things so she would have no problem with this.

Mother of a Hispanic White female child aged 9 years

###### Session Reminder Feature

The caregivers had multiple complaints about this feature. They felt that the session reminder feature was difficult to set because the settings icon, where this feature is housed, was challenging to find and should be more prominent. They did not like housing this feature within the settings tab. A suggested alternative location was the home screen. The caregivers did not like the color of the current settings icon (ie, a gray gear image). They recommended changing the current gear image to an alarm, a bell, or a clock image.

##### Preferences

This organizing theme summarized the caregivers’ critiques of the overall app’s esthetics and guided imagery session predilections. It also captured the caregivers’ suggestions for incentives for encouraging their children to use the GIT app regularly.

###### GIT App Interface

The caregivers made numerous comments about the app’s esthetic design, including its font, background, and images. They thought that the current font size was too small. They conveyed that the pictures and icons were satisfactory and that they liked the current color scheme. Moreover, they thought that on the log-in screen, the size of the images should be increased and both the fields and images should be centered:

But then, yes, all the little, like the home, the back button, the settings, that’s very, very little.

Mother of a non-Hispanic White male child aged 8 years

Finally, the caregivers disagreed on whether the GIT app session player screen image should be animated. Although some caregivers advocated for images or cartoons of the guided imagery session on the player screen, most caregivers did not recommend this approach:

I would say no, honestly, because if you’re...unless that’s what you’re wanting to do. If you’re wanting it to be that I’m going to watch a video, like we use distractions of video games and all that kind of stuff, then that’s a whole different thing than saying I want you to, like a mindfulness type, yoga, like us, let me just relax, meditate. Otherwise, they’re going to watch the screen. And so if you’re really going for the meditation, this needs to be as plain as possible, so they don’t look at it. That’s just my thought.

Mother of a non-Hispanic White male child aged 11 years

###### GIT Sessions

The caregivers liked the sample session and the overall approach to treating children with FAPDs. However, they thought that the snow sledding audio session did not align with the snowflake image on the main audio track screen.

They provided insight into preferred session topics that would be ideal for their children. They recommended beach activities such as surfing and session settings that include animals such as dogs, cats, and unicorns. They also suggested session settings such as forests, boating, and biking adventures and the inclusion of superhero characters in the narrative.

They thought that some session characteristics should be avoided. Scary animals (eg, sharks, snakes, spiders, tarantulas, and insects), violence, and natural catastrophic events such as earthquakes or flooding were not recommended. Anything frightening, such as war, fighting, darkness, monsters, and spooky ghosts, was not recommended as session settings. They did not recommend having a “bad guy” or an antagonist role in a session.

They suggested adding sounds to the sessions. The proposed sounds varied but included classical or instrumental music, nature sounds (eg, ocean waves, rain, babbling brook, waterfall, wind, and outdoor sounds), and sounds related to the session setting. There was no consensus on whether a male or female voice was preferred for session narration.

The caregivers commented on the ideal session duration; however, similar to what was observed with children, no clear consensus was reached. The caregivers thought that the 8-minute-and-30-second sample session was acceptable and that 10- to 15-minute sessions would be the longest tolerated by children. Some caregivers were concerned that longer sessions would not retain their children’s attention and may make their children reluctant to complete future sessions. In addition, a few caregivers were concerned that their children would be unable to use their imagination for longer than 8 minutes.

###### Incentives for Promoting Routine Use

The caregivers were informed that children with FAPDs would ideally have to listen to a session almost daily to treat their abdominal pain. The caregivers thought that parental supervision would promote adherence to the app and that tracking the level of abdominal pain before and after the session, thus highlighting the improvements thereof, would motivate children to use the app. However, they also thought that repetitive use of the app would decrease children’s motivation over time.

The caregivers also expressed that intangible and tangible rewards should be used to increase routine engagement with the app. They thought that intangible rewards such as tokens, points, badges, trophies, stickers, stars, and stamps would be ideal incentives for listening to the sessions in the app. They also liked the idea of rewarding children with money or gift cards. Finally, they thought that rewarding children with an electronic game inside or outside the app would be preferred.

### Integration

Collectively, the qualitative and quantitative results revealed favorable usability. The SUS survey indicated that the GIT app has above-average usability as rated by the children and their caregivers; however, areas that could be improved to further enhance usability were identified. The software evaluations provided further insight into the usability challenges faced by the children and their caregivers by highlighting the consistent problem of locating the reminder notification feature. Qualitative findings from the interviews with the children and their caregivers confirmed the usability issues related to locating the reminder feature and revealed that its placement in the settings tab denoted by a gear symbol was not intuitive. In addition, complementary factors related to the app’s esthetics (eg, small fonts and images and nonintuitive images used as icons) may have also impacted the usability of the app prototype.

## Discussion

### Overview

The ubiquity of personal electronic mobile devices such as smartphones in our society, regardless of socioeconomic status, presents a unique opportunity to deliver medical and mental health therapy for various diseases and conditions in a nontraditional manner [29]. The potential impact of our GIT app on treating children with FAPDs remotely would overcome substantial barriers to accessing optimal care, such as limited access to trained providers and frequent in-person sessions [30,31]. We used the resource-intense user-centered design approach to evaluate the GIT app’s usability, increase its overall appeal for routine use, and hopefully decrease the need for long-term technical assistance resources [16,17,32]. Our study used qualitative and quantitative research to capture feedback from young pediatric patients with FAPDs and their caregivers about our GIT app prototype to inform our future refinement process.

### Principal Findings and Comparison With Prior Work

Despite other web-based interventions designed to treat FAPDs reported in the literature, this study, to our knowledge, is the first to report the critiques of an electronic therapy intervention from children with FAPDs and their caregivers. Our mixed methods approach used quantitative and qualitative data to assess our GIT app’s usability and captured children’s and caregivers’ preferences for our current prototype. Furthermore, the children and their caregivers offered information about rewards and how incentives may encourage their regular use of the app and maximize its therapeutic benefit. Domhardt et al [33] conducted a systematic review to assess the quality of pediatric mobile apps targeting anxiety, depression, and posttraumatic stress disorder. They found 15 apps that offered interventions with cognitive behavioral therapy elements, relaxation exercises, mindfulness, psychoeducation, and physical exercises, which are similar interventions for pediatric FAPDs. One of the apps incorporated gamification techniques. However, only one of these apps was tested in a randomized controlled trial. The authors also reported that these reviewed mobile apps had poor quality and “an absence of scientific‑driven development and lack of methodologically sound evaluation of apps” [33]. Opportunities to increase access to care among pediatric patients with FAPDs and those with mental health disorders via mobile apps exist; the development of such apps need to be informed by the needs and participation of children and caregivers. However, more investment in mobile app development (as in this study) and efficacy testing is needed to deliver a quality therapeutic product to these vulnerable patient populations.

Our study was informed by Zhao et al [34], which outlines the vital elements that promote health behavior change through effective mobile apps, including user-friendly design and personalized features. We directly addressed the need for a user-friendly design by adopting a mixed methods approach before and during app development. Our SUS results show that the children and their caregivers ranked the GIT app’s usability between “good” and “excellent” and that the app had acceptable reliability. Our current investigation also discovered a reproducible usability issue that occurred when setting up the reminder notification feature inside the app. This reminder feature will be modified in future iterations, given its known role in promoting adherence for chronic pain conditions [35].

The interview results led to the exploration of the GIT app’s esthetics and guided imagery sessions. The children’s and caregivers’ preferences for session settings and incentive rewards reported in this study were similar to those reported in our predevelopment paper [14]. These similarities reinforce the need to develop engaging sessions that children will want to listen to with minimal prompting. We also discovered the value of achievement-based intangible rewards such as electronic trophies and coins that would motivate children to engage with the GIT app; exploiting gamification principles within this app appears to be an ideal technique for this pediatric patient population with FAPDs [36].

The exploration of personalized features for the GIT app led to requests for visual animations, additional music and sounds during session playback, and short session durations. However, we are concerned that these preferences may compromise the efficacy of the sessions. Guided imagery requires narration to create “mental images that bring about a state of focused concentration” that leads to relaxation and subsequent “physical and emotional well-being” [37]. Presenting distracting visual images or external audio sounds during sessions could impair the mental processing of the therapy narration, hinder the creation of imaginary images, and possibly dilute the treatment effect. This ambiguity must be addressed in future studies.

Furthermore, adequate time is required during a session to become fully engaged in GIT. In a previous study that used audio recorded guided imagery to treat pediatric FAPDs, exit interviews revealed that the sessions (which lasted for approximately 10 to 25 minutes) were enjoyable, children did not need to be prompted by their parents to listen to the sessions, and most children listened to the sessions more often than instructed [11]. This feedback suggests that children and their caregivers may underestimate the importance of engagement during GIT when listening to a brief audio sample rather than an entire session. Future GIT app intervention implementation should communicate to children and their caregivers the overall goals of the sessions and teach them how to best create an ideal environment for conducting sessions at home. Child psychology expert opinion is needed to decide whether additional audio sounds should be incorporated into future sessions without compromising their therapeutic effect before efficacy testing.

### Limitations and Strengths

Despite our rigorous approach to assessing the GIT app, our study’s results have limitations. The transferability of our results to other therapeutic mobile apps may be applicable only in a similar context. Of note, our sample included younger children with FAPDs and their caregivers who live in the Southwest United States, and caution should be taken when applying the findings of this study to circumstances involving other age groups, medical conditions, geographic areas, cultures, etc [38].

The strengths of our study include the use of both quantitative and qualitative methods to enhance the study’s rigor to gain greater insight into the GIT app’s usability. Collecting data from both parents and children is also a strength, in that we will be able to enhance app’s usability based on the feedback from the 2 key stakeholders. In addition, the qualitative interviewers were trained to use probes, prompts, and clarifying statements to ensure that the voices of the participants were captured during the interviews [39]. We were able to translate our SUS scores into an adjective rating scale that is relatable to readers who are not familiar with human factor research. Finally, we reached saturation in our qualitative data collection, meaning that no new information emerged when we stopped the interviews.

### Conclusions

Incorporating a user-centered design into our development process has revealed multiple recommendations from children and caregivers for improving our GIT app. The children with FAPDs and their caregivers rated our novel GIT app as having above-average usability overall; however, usability can be improved by addressing the issue with the location of the reminder notification feature within the app. These dyads also provided vital information to improve the app’s esthetics, tailor its sessions for pediatric patients with FAPDs, and encourage routine use through gamification techniques. This information lays the foundation for our continued development of a user-centered, therapeutic mobile health app targeted explicitly for this vulnerable patient population with chronic pain.

## Appendix

Multimedia Appendix 1 Software evaluation task list.

Multimedia Appendix 2 Child Systems Usability Scale.

Multimedia Appendix 3 Caregiver Systems Usability Scale.

Multimedia Appendix 4 Child interview script.

Multimedia Appendix 5 Caregiver interview script.

## Abbreviations

ARGI: Audio Recorded Guided Imagery
FAPD: functional abdominal pain disorder
GIT: guided imagery therapy
SUS: System Usability Scale

## Data Availability

The data generated during or analyzed during this study are available from the corresponding author upon reasonable request.
