# Supplementary material for: Children’s and Caregivers’ Review of a Guided Imagery Therapy Mobile App Designed to Treat Children With Functional Abdominal Pain Disorders: Leveraging a Mixed Methods Approach With User-Centered Design
Source: JMIR Form Res. 2023 Apr 19;7:e41321. doi: 10.2196/41321 (PMC10157463; doi:10.2196/41321)
Supplement: Multimedia Appendix 1 [file formative_v7i1e41321_app1.docx]

Argit Task List

Please place a 🗷 or 🗹 when you have completed each task. Thank You!

- Open the Argi app
- Log In

Email: [belly@abcd.com](mailto:belly@abcd.com)

Password: 12345

- Open the Audio Track
- Choose the Snow Session
- Go back to the previous screen
- Set a reminder notification to 7:30pm
- Exit out of the app
