# Supplementary material for: Children’s and Caregivers’ Review of a Guided Imagery Therapy Mobile App Designed to Treat Children With Functional Abdominal Pain Disorders: Leveraging a Mixed Methods Approach With User-Centered Design
Source: JMIR Form Res. 2023 Apr 19;7:e41321. doi: 10.2196/41321 (PMC10157463; doi:10.2196/41321)
Supplement: Multimedia Appendix 2 [file formative_v7i1e41321_app2.docx]

APPENDIX A. Child System Usability Scale

|  | Strongly Disagree | Disagree | Neither Agree or Disagree | Agree | Strongly Agree |
| --- | --- | --- | --- | --- | --- |
| 1. I think that I would like to use this mobile app a lot | ◯ | ◯ | ◯ | ◯ | ◯ |
| 1. I found the mobile app was hard to use | ◯ | ◯ | ◯ | ◯ | ◯ |
| 1. I thought the mobile app was easy to use | ◯ | ◯ | ◯ | ◯ | ◯ |
| 1. I think that I would need someone to help me use this mobile app | ◯ | ◯ | ◯ | ◯ | ◯ |
| 1. I found the various parts of this mobile app worked well together | ◯ | ◯ | ◯ | ◯ | ◯ |
| 1. I thought it was hard to figure out how to do certain things on the mobile app | ◯ | ◯ | ◯ | ◯ | ◯ |
| 1. I think that most people would learn to use this mobile app very quickly | ◯ | ◯ | ◯ | ◯ | ◯ |
| 1. I found the mobile app very hard to use | ◯ | ◯ | ◯ | ◯ | ◯ |
| 1. I felt very confident using the mobile app | ◯ | ◯ | ◯ | ◯ | ◯ |
| 1. I needed to learn a lot of things before I could get going with this mobile app | ◯ | ◯ | ◯ | ◯ | ◯ |
