# Supplementary material for: Children’s and Caregivers’ Review of a Guided Imagery Therapy Mobile App Designed to Treat Children With Functional Abdominal Pain Disorders: Leveraging a Mixed Methods Approach With User-Centered Design
Source: JMIR Form Res. 2023 Apr 19;7:e41321. doi: 10.2196/41321 (PMC10157463; doi:10.2196/41321)
Supplement: Multimedia Appendix 3 [file formative_v7i1e41321_app3.docx]

APPENDIX B. Caregiver System Usability Scale

|  | Strongly Disagree | Disagree | Neither Agree or Disagree | Agree | Strongly Agree |
| --- | --- | --- | --- | --- | --- |
| 1. I think that I would like to use this mobile app frequently | ◯ | ◯ | ◯ | ◯ | ◯ |
| 1. I found the mobile app unnecessarily complex | ◯ | ◯ | ◯ | ◯ | ◯ |
| 1. I thought the mobile app was easy to use | ◯ | ◯ | ◯ | ◯ | ◯ |
| 1. I think that I would need the support of a technical person to be able to use this mobile app | ◯ | ◯ | ◯ | ◯ | ◯ |
| 1. I found the various functions in the mobile app were well integrated | ◯ | ◯ | ◯ | ◯ | ◯ |
| 1. I thought there was too much inconsistency in this mobile app | ◯ | ◯ | ◯ | ◯ | ◯ |
| 1. I would imagine that most people would learn to use this mobile app very quickly | ◯ | ◯ | ◯ | ◯ | ◯ |
| 1. I found the mobile app very cumbersome to use | ◯ | ◯ | ◯ | ◯ | ◯ |
| 1. I felt very confident using the mobile app | ◯ | ◯ | ◯ | ◯ | ◯ |
| 1. I needed to learn a lot of things before I could get along with this mobile app | ◯ | ◯ | ◯ | ◯ | ◯ |
