# Supplementary material for: Children’s and Caregivers’ Review of a Guided Imagery Therapy Mobile App Designed to Treat Children With Functional Abdominal Pain Disorders: Leveraging a Mixed Methods Approach With User-Centered Design
Source: JMIR Form Res. 2023 Apr 19;7:e41321. doi: 10.2196/41321 (PMC10157463; doi:10.2196/41321)
Supplement: Multimedia Appendix 4 [file formative_v7i1e41321_app4.docx]

Appendix C. Child Interview

Interviewer: Thanks for talking with me today. Now that you have just used the mobile app, I have some questions for you. Some of my questions may be hard to answer but please give me your honest opinion even if negative. Our goal is to improve the app so kids can use this app to fix their belly pain.

1. What do you think about using the app to treat your belly pain? (Prompt for desire to want to use it or not, capture strengths and how to improve)

1. Earlier we asked you to do a number of tasks with the app like logging into the program. Were any of these tasks easy to do? Were any of these tasks difficult to do? (Prompt: Based on a scale from 1 to 3 with 3 being the most difficult, tell me about logging in, forgot password retrieval, finding study research coordinator contact information, accessing the guided imagery session menu, initiating guided imagery sessions with the audio player; Probe for ways to make these process less difficult)

1. When you used the app, did it respond to your finger commands as expected? (Probe for imperfections related to specific tasks with the app if the response is negative)

1. Did you discover any glitches or errors with the mobile app while you were using it?

1. On a scale of 1 to 3, with 1 being too easy, 2 being just right, and 3 being too hard, how comfortable are you with using this mobile app? (Probe for both strengths and weakness of the app)

****We plan to probe the respective questions if the participant scores any item poorly on the System Usability Scale****

1. What do you expect this app and the guided imagery sessions to do for you?
2. What do you think about the app’s appearance? (Prompt for general topics about the app’s icons/pictures, background colors, and font style, size, and color, Probe for reasons about negative comments pertaining to these topic)
3. What do you think about the font? (Probe for font type and size)
4. What do you think about logging into the app? (Probe for ease and/or difficulty, probe into how can we improve the process)

1. What are some other options you would prefer in order to log into the app? (Prompt for use of avatar, using a code vs. password)

1. Sometimes people forget their passwords. What is the best way to receive this app’s password if you or your caregiver forget? Why? (Prompts can include email, text message, phone calls, etc.)
2. How well do you think the app works? (Prompt: ease of finding the guided imagery session selection menu, initiating a guided imagery session on the audio player, set a reminder)

1. The session you just heard about was snow. What did you think about this session? (Probe for thoughts about the speaker. Explore preference of male vs. female voice.)

1. What are your thoughts about picture used during the session? (Probe for the use of visual components such as real life vs. graphic image and still image vs. animation
2. How many sessions would you suggest be available on the app?

1. The guided imagery session you just heard was about snow and it is designed to help you relax. What other guided imagery session topics would you like hear to help you relax?

1. What are your thoughts on the use of background sound during the session? (Probe for music and sound correlating with the session topic.)

1. What guided imagery session topics would you not want to hear when you relax? (Prompt for scary, repulsive topics, Probe about colors of the app in the app)
2. We would need you to listen to a session almost every day to help your belly pain. The guided imagery session you just heard was about 8 minutes. On a scale of 1 to 3, with 1 being too short, 2 being just right, and 3 being too long, what do you think about the length of the session you just heard? (Probe into reasons for their respective response. Prompt to assess how long future sessions should be and probe for reasons for their response.)

1. We would like you to listen to a guided imagery session almost every day. What would help to remind you to do the sessions every day? (Prompt for reminders given by the app and how it would be delivered within the app—pop-up notifications, audible alert, email reminder, etc.; prompt for utility of possible rewards and type of rewards to encourage consistent use)

1. Where would be the best place to put the reminder notification?

1. What are some other types of reminders would you suggest? (Prompt for thoughts on about pop-up notifications and text notifications. Probe to see which one they would prefer.)

1. What would motivate or inspire you to do the sessions? (Probe: rewards, award types, electronic trophies, points. Probe for previous thoughts of using progress reports before/after sessions.)
