# Supplementary material for: Children’s and Caregivers’ Review of a Guided Imagery Therapy Mobile App Designed to Treat Children With Functional Abdominal Pain Disorders: Leveraging a Mixed Methods Approach With User-Centered Design
Source: JMIR Form Res. 2023 Apr 19;7:e41321. doi: 10.2196/41321 (PMC10157463; doi:10.2196/41321)
Supplement: Multimedia Appendix 5 [file formative_v7i1e41321_app5.docx]

Appendix D. Caregiver Interview

Interviewer: Thanks for participating with this study. Now that you have just finished using our mobile app, I have some questions regarding how well it works. Our goal is to improve the app in order to engage children in using this app to treat their belly pain. Some of my questions may be difficult to answer but please give me your honest opinion even if negative.

1. What do you think about using the app to treat your child’s belly pain? (Prompt for desire to use or not, capture strengths and how to improve)

1. Earlier we asked you to do a number of tasks with the app like logging into the program. Were any of these tasks easy to do? Were any of these tasks difficult to perform? (Prompt: Based on a scale from 1 to 3 with 3 being the most difficult, tell me about assessment of logging in, forgot password retrieval, finding study research coordinator contact information, accessing the guided imagery session menu, initiating guided imagery sessions with the audio player; Probe for ways to make these process less difficult)

1. When you used the app, did the app respond to your finger commands as expected? (Probe for imperfections related to specific tasks with the app if their response is negative)

1. Did you discover any glitches or errors with the mobile app while you were using it?
2. On a scale of 1 to 3, with 1 being too easy, 2 being just right, and 3 being too hard, how comfortable are you with using this mobile app? (Probe for both strengths and weakness of the app)

****We plan to probe the respective questions if the participant scores any item poorly on the System Usability Scale****

1. What do you expect the app or guided imagery sessions to do for your child?
2. What do you think about the app’s appearance? (Prompt for general topics about the app’s icons/pictures, background colors, and font style, size, and color, Probe for reasons about negative comments pertaining to these topics
3. What do you think about the font? (Probe for font type and size)
4. What do you think about logging into the app? (Probe for ease and/or difficulty, capture positive aspects, probe into how can we improve the process)

1. What are some other options you would prefer in order to log into the app? (Prompt for use of avatar, using a code vs. password)
2. Sometimes people forget their passwords. What is the best way to receive this app’s password if you and your child forgets? Why? (Prompts can include email, text message, phone calls, etc.)

1. What do you think about the operation of the app? (Prompt: ease of finding the guided imagery session selection menu, initiating a guided imagery session on the audio player)

1. The session you just heard about was snow. What did you think about the session? (Probe for thoughts about the speaker. Explore preference of male vs. female voice.)

1. What are your thoughts about the picture used during the session? (Probe for the use of visual components such as real life vs. graphic image and still image vs. animation)

1. How many sessions would you suggest be available on the app?
2. The guided imagery session you just heard was about snow and it is designed to help your child relax and manage their abdominal pain. What other topics of guided imagery sessions would your child like to hear?
3. What are your thoughts on the use of background sound during the session? (Probe for music and sounds correlating with the session topic.)

1. What topics of guided imagery session would your child not want to listen? (Prompt for scary, repulsive topics)

1. For guided imagery therapy, we would need your child to listen to a session almost every day to be effective. The guided imagery session you just heard was about 8 minutes. On a scale of 1 to 3, with 1 being too short, 2 being just right, and 3 being too long, what do you think about the length of the session you just heard for your child? (Probe into reasons for their respective response. Prompt to assess how long future sessions should be and probe for reasons for their response; explore the option of ( building up to 15 min - for example, start with 5, then over a few sessions get to 15; or what about giving them the opportunity to choose the amount of time, ranging from 5 - 30 min)

1. Sometimes it can be hard to remember to do a guided imagery session every day. What could we do to help you remember to use the app? (Prompt for reminders given by the app and how it would be delivered within the app—pop-up notifications, audible alert, email reminder, etc.; prompt for utility of possible rewards to encourage consistent use)

1. Where would be the best place to put the reminder notification?

1. What are some other reminder methods you would recommend? (Prompt for thoughts on about pop-up notifications and text notifications. Probe to see which one they would prefer.)
2. Is there anything that we can do to motivate your child to use this app? (Probe: rewards, award types, electronic trophies, points***.*** Prompt the previous thoughts of using progress reports before/after sessions.)
